# A study of SPECT/CT camera stability for quantitative imaging

**DOI:** 10.1186/s40658-016-0150-7

**Published:** 2016-07-29

**Authors:** Wendy A. McDougald, Robert S. Miyaoka, Adam M. Alessio, Robert L. Harrison, Thomas K. Lewellen

**Affiliations:** Imaging Research Laboratory, Department of Radiology, University of Washington, 222 Portage Bay Building, Box 357987, Seattle, WA 98195-7987 USA

**Keywords:** Single photon emission tomography with computer tomography (SPECT/CT) quantitation, Conversion factor, SUV accuracy

## Abstract

**Background:**

The purpose of this study was twofold: to evaluate the quantitative stability of a SPECT/CT gamma camera over time and to determine if daily flood acquisitions can reliably serve as calibration factors for quantitative SPECT. Using a cylindrical water phantom filled with measured amounts of ^99m^Tc, factors were calculated to convert counts/cc to activity/cps. Measurements were made over an 18-month period. System sensitivity data calculated from ^57^Co daily quality assurance (DQA) flood acquisitions were then compared to the ^99m^Tc calibration factors to determine the relationship of the factors.

**Results:**

The coefficient of variation is 2.7 % for the ^99m^Tc cylinder conversion factors and 2.6 % for the ^57^Co DQA flood data. The greatest difference between the cylinder conversion factors and the flood data is less than 3 %.

**Conclusions:**

Based on the results, the camera was stable within 3 % over an 18-month time period. The daily flood source acquisitions can be a reliable source for tracking camera stability and may provide information on updating the calibration factor for quantitative imaging.

## Background

For years, the concept of single photon emission tomography (SPECT) and now, SPECT integrated with X-ray computerized tomography (SPECT/CT) being used as a quantitative imaging tool has been pursued. Recently, partially due to improved technologies and vendor interest, fully quantitative SPECT/CT as a standard clinical tool is close to becoming a reality. While positron emission tomography with X-ray computerized tomography (PET/CT) has seen increased usage as a quantitative diagnostic and staging tool, one drawback is that it is limited to only a few FDA-approved radiopharmaceuticals. SPECT/CT is advantageous as a fully quantitative tool since it can be performed with a substantially larger list of approved radiopharmaceuticals.

With this in mind, research continues on the accuracy/validation of SPECT/CT image reconstruction, scatter correction, collimator response, and attenuation correction. For example, several studies have assessed quantitative SPECT/CT for ^111^In agents [1–3], ^99m^Tc in lung perfusion, ^201^Tl in myocardial perfusion [4, 5] and brain imaging [6], ^123^I for DAT scans [7], or ^177^Lu and ^131^I for dosimetry calculations [8, 9].

However, in attempting to obtain quantitative information from images, such as the standard uptake value as used in PET/CT, one factor to first consider is the stability of the SPECT/CT camera. The overall consistent performance of the camera becomes an important issue regarding the accuracy, precision, or even the reproducibility of clinical trials or serial studies. If the goal of activity quantification is to enable diagnostic or therapeutic decisions based on estimates of activity in objects or regions in the body [10], then not only the individual components need scrutiny but the camera’s performance as a whole unit needs to be evaluated for continuity and consistency.

In order to investigate the camera’s stability, we obtained images of the same cylindrical phantom filled with a known amount of activity approximately once a week over an 18-month period. This allowed us to analyze the accuracy and precision of scanner-estimated activity concentrations for numerous data points over that time period. It also allowed us to more closely evaluate issues that arose, identifying sources of potential errors or biases.

Manufacturers recommend obtaining flood phantom images of a sheet source for daily quality assurance (DQA). For the camera used in this study, we evaluated daily ^57^Co flood phantom images taken as part of our clinic’s DQA. The DQA scans were acquired over the same time period as the cylindrical phantom studies were reviewed. The detection efficiency for the DQA scans were analyzed and compared to the cylindrical phantom results, noting any time varying behavior or scanner variability.

## Methods

To evaluate the stability of the SPECT/CT scanner and determine a calibration factor for quantitative SPECT/CT, the measured activity concentration of a water-filled phantom injected with a radionuclide along with the counting rate from the DQA ^57^Co flood phantom was measured and tracked for approximately a year and a half. The study was done on a Philips Precedence, dual-head SPECT with a 16-slice helical CT system (Philips Healthcare, Cleveland, OH).

Vendor-recommended quality assurance testing on the Philips Precedence was conducted on a daily basis prior to scanning. For monitoring the extrinsic integral and differential uniformity, the DQA was completed on the gamma camera detector component using the ^57^Co flood phantom. The camera’s spatial resolution, linearity, and the center of rotation (COR) were extrinsically tested weekly. The CT component’s DQA requirements were also conducted prior to scanning. This process required scanning the manufacturer’s CT quality control phantom for verification of Hounsfield units and visually inspecting the images for uniformity and artifacts. The Philips Precedence SPECT/CT system is accredited by the American College of Radiology Imaging Network (ACRIN), which requires passing extensive annual quality assurance testing on the SPECT and CT components.

Scans of all the phantoms were performed using a basic patient torso protocol: step and shoot parameters of 20s per view, 128 × 128 matrix, non-circular orbit, 64 views over 180°; and the accompanying helical CT image: 120 kVp, 50 mAs, and 0.94 pitch. An energy window of 20 % centered around 140 keV (energy window peak checked/centered for each acquisition), low energy effectivity at 126 keV, and high energy effectivity at 154 keV was used.

The low-energy high-resolution (LEHR) collimator was used for all the ^99m^Tc-filled phantoms and the majority of the ^57^Co flood phantoms. Occasionally, a medium- or high-energy general purpose collimator was used during the acquisition of the DQA flood scan: for consistency, these have not been used in our analysis.

In order to consider possible attenuation and scatter correction biases, some scans were performed with adipose tissue equivalent material (TEM; which has absorption and scattering properties within 1 % of living tissue)—3 cm thick, 30 × 30 cm^2^ wrapped around the 20-cm cylinder phantom.

### Phantoms

1. Twenty-centimeter-diameter right circular cylinder phantom, volume of 6730 cc, filled with water injected with 10 mCi [9–11 ± 1 mCi] of ^99m^Tc
2. NEMA image quality phantom, volume 10,490 cc, filled with water injected with 1 mCi of ^99m^Tc
3. ACR SPECT phantom, volume 5743 cc, water filled, injected with 5 mCi of ^99m^Tc
4. One-, two-, or four-liter bottle, water filled and injected with 5 mCi of ^99m^Tc
5. National Institute of Standards and Technology (NIST) traceable standard ^57^Co solid phantom 20 mCi flood source, with an activity matrix of 16.25 ± 0.04 by 23.92 ± 0.04 in.

**Fig. 1:**
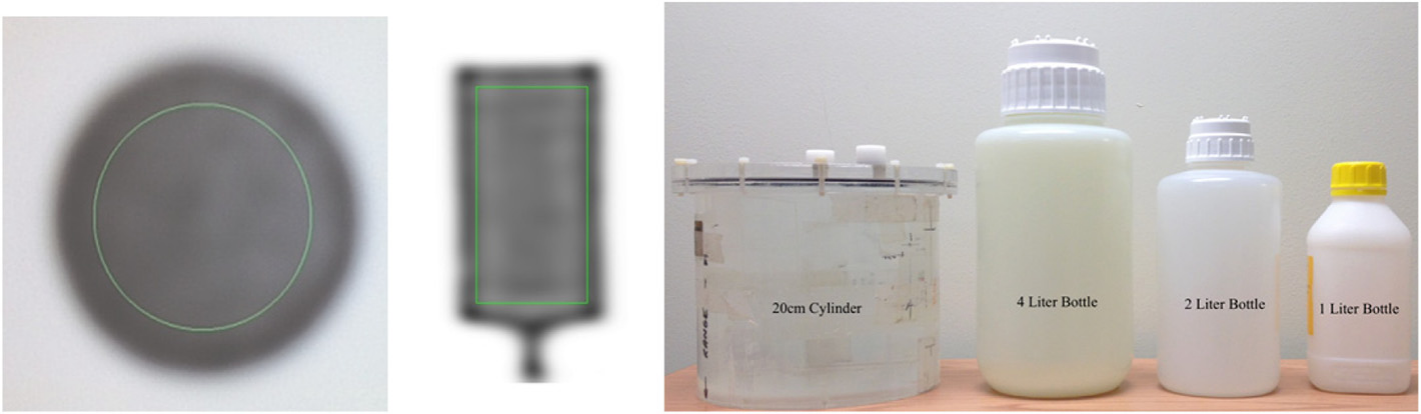
Example of VOI drawn on the cylinder phantoms and the liter bottles along with images of the main phantom used, 20-cm cylinder, 4-, 2-, and 1-L bottles

Images of the liter bottles and the 20-cm right cylinder phantom are seen in Fig. 1.

### Reconstruction method

The vendor-supplied Astonish ordered subsets expectation maximization (OSEM) iterative algorithm was used; it incorporates depth-dependent resolution recovery, attenuation, and scatter correction [11, 12]. For scatter correction, Astonish uses a version of the effective source scatter estimation (ESSE) method which corrects for scatter by first estimating an effective scatter source that is then projected using an attenuated projection operation [13]. The OSEM reconstruction was done with six iterations and eight subsets and with a Hanning filter with a cutoff frequency of 1 included in the reconstruction loop.

### ^99m^Tc phantom data

After injecting a phantom with a measured amount of ^99m^Tc, it was scanned using the standard patient protocol mentioned above. The collimator/detector heads are moved in as close as possible to the phantom, with a radius range of 24 cm right and left lateral, 20 cm anterior, and 22 cm posterior. A volume of interest (VOI) was drawn on the reconstructed image in order to obtain counts per second per volume (cc). The activity concentration (AC) was calculated based on the decay-corrected activity per phantom volume (cc) in units of cps per microcurie as shown in Eq. 1.

The conversion factor (CF) was calculated as the ratio of the known activity concentration (*A*) in the phantom to the measured counts per volume (*M*). Specifically, a VOI was drawn on the phantom (see example in Fig. 1); the numerator, *A*, is the known activity concentration based on the injected activity and phantom volume. The average of the measured counts per second per volume (cps/cc) of the VOI is the denominator in Eq. 1, (M). The CF is calculated as follows:

1 $$ \mathrm{C}\mathrm{F}\ \left[\frac{\upmu \mathrm{C}\mathrm{i}}{\mathrm{cps}}\right]=\frac{A\left[\frac{\upmu \mathrm{C}\mathrm{i}}{\mathrm{cc}}\right]}{M\left[\frac{\mathrm{cps}}{\mathrm{cc}}\right]} $$

### ^57^Co flood phantom data

The DQA scans were taken with the manufacturer’s recommended protocol; acquiring 15 million counts per detector head, energy window of ±10 % at 122 keV, with the NIST ^57^Co flood phantom placed directly on detector head 2 and detector head 1 positioned close to the phantom.

The ^57^Co flood phantoms were purchased from the same manufacturer, with a measured activity of 20 mCi and a guaranteed <0.08 % combined ^56^Co/^58^Co radionuclide impurity. As the ^57^Co decays, different flood phantoms are used for the DQA. Depending on the ^57^Co activity level in the flood phantom, floods may be used separately or in combinations with other older flood phantoms. Therefore, during the study, three different ^57^Co flood phantoms were used for the DQA, as noted in Table 1. For example, the 2010 and 2011 phantoms were used together after the 2011 phantom’s ^57^Co activity level became too low.

**Table 1:** Time periods for the different DQA ^57^Co flood phantoms used over the course of the study

^57^Co flood phantom’s activity source reference year	Time period flood(s) used
Flood A: 2011	5/2/2012–7/9/2012
Flood B: 2010 + 2011	7/26/2012–10/24/2012
Flood C: 2011 + 2012	12/19/2012–5/2/2013

It should be noted that the ^57^Co flood phantoms are used consistently every weekday morning for the DQA throughout the year. The time periods shown above only reflect times that directly coincide with when the 20-cm phantom or liter bottle scans were performed.

All the ^57^Co flood data were decay corrected using the standard half-life of 271.9 days for ^57^Co. Total counts per second from each detector head, with an area of approximately 54,500 mm^2^, were recorded.

Counts per second from the DQA ^57^Co flood scans taken on the same days as the ^99m^Tc scans were used for the comparison with the conversion factors. For each day, the flood sensitivity (FS) was calculated using the mean of detector 1 plus detector 2 counts, then decay corrected. The decay-corrected values were then normalized to the mean of flood A, then to 1 for scaling. The conversion factor values were divided by the flood sensitivity values (CF/FS) and compared to the conversion factor values, in order to assess the use of DQA data to compute the calibration factor.

**Fig. 2:**
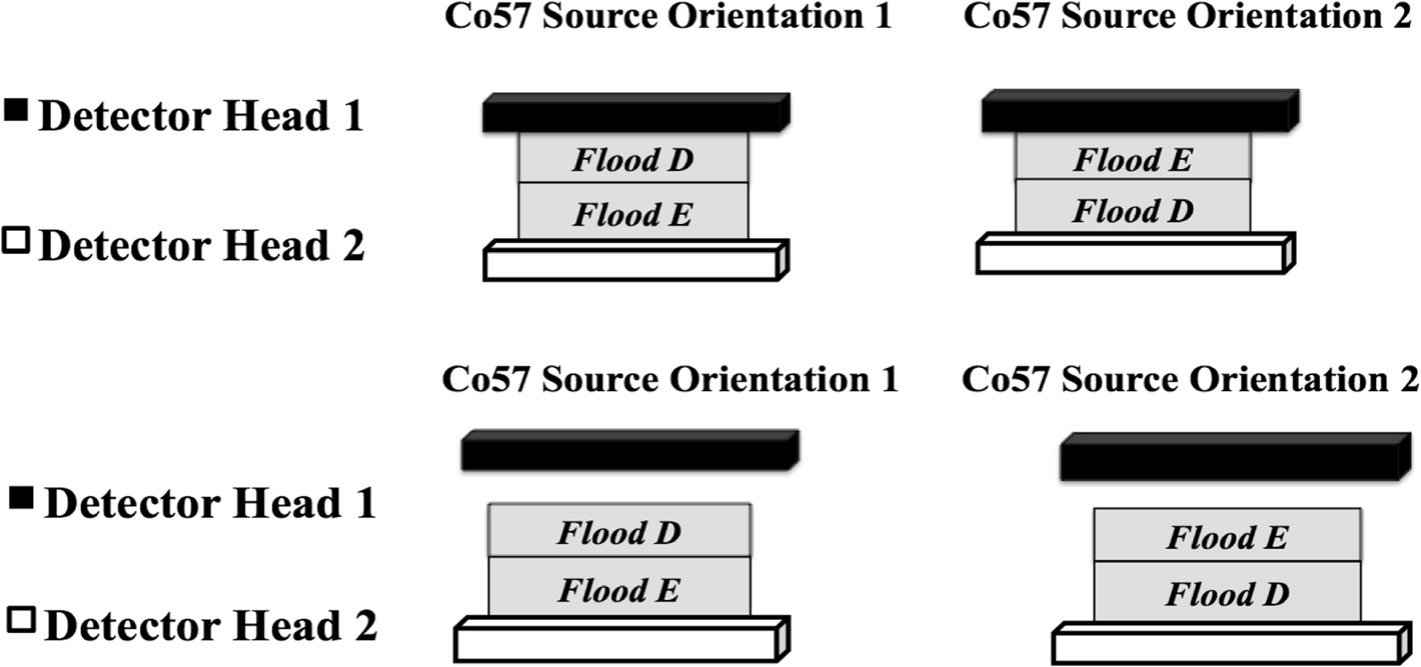
Placement of flood D and flood E on detector heads 1 and 2 and placement of flood D and flood E on detector heads 1 and 2, with detector 1 moved away from the flood phantoms

A second, separate, DQA flood study was done for comparison with flood phantom B. This involved using two new ^57^Co flood phantoms of different activity levels of ^57^Co labeled: flood D dated 2012 and flood E dated 2013. Three sets of data were collected during the DQA using floods D and E with two different position/stacking orientations (1 and 2) and one different detector position as shown is Fig. 2.

### CT images

The vendor’s software generates an attenuation map, per pixel value, from the CT images. Based on the CT Hounsfield (HU) numbers, the values are converted to the linear attenuation coefficients. Therefore, regions of interest (ROIs) where drawn on several of the corresponding CT images for a visual inspection, validation, and stability of the HU numbers.

## Results

### Precedence ^99m^Tc conversion factors

**Fig. 3:**
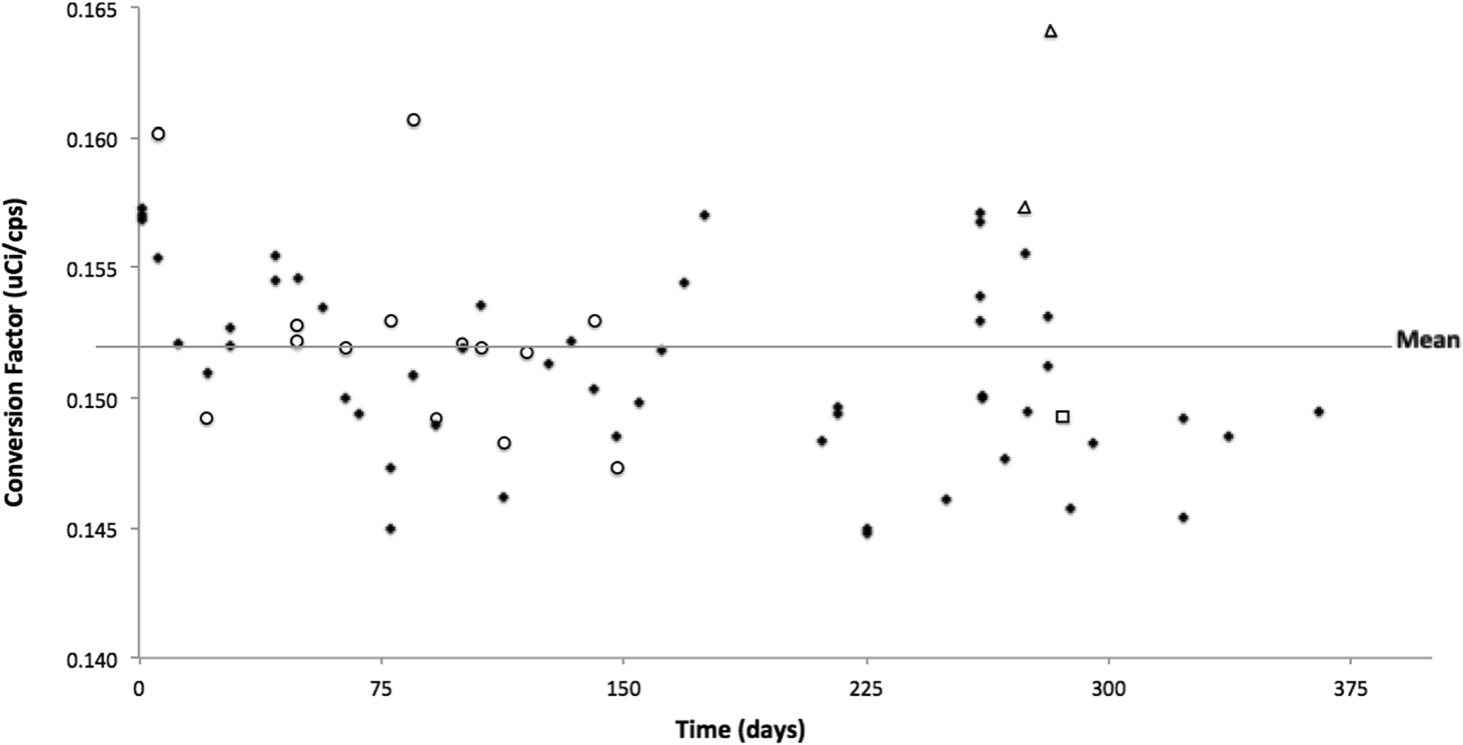
Graph of the conversion factors for ^99m^Tc complete data set over the entire 18 months of the study. Data set includes all phantoms, 20 cm, ACR, and IQ (*solid diamond*) and all liter bottles: 1 (*circle*)-, 2 (*triangle*)-, and 4 (*square*)-L. 72.5 % of the data points are within one standard deviation of the mean

Figure 3 shows the distribution of activity conversion factors calculated using a ^99m^Tc source scanned on the Philips Precedence SPECT/CT gamma camera. The graph displays the results generated from the various phantoms and geometries used: the 20-cm cylindrical phantom with and without 3 cm of TEM material added; the ACR and IQ cylinder phantoms; along with the 1-, 2-, and 4-L bottles.

As seen in Fig. 3, during the first couple of months of the study, the conversion factors remained just above the mean, and then started to drift below. Calibrations and preventive maintenance (PM) were performed on the scanner on 5 October 2012, after which there was a decrease of approximately 4 % in conversion factors derived from the cylindrical phantom for that month. The conversion factors then began a slight shift upward. Prior to and up to the PM, there was an overall increase in the conversion factors of 4 %. In April of 2013, the center of rotation calibration (COR) and adjustments were made along with a second PM. Following the COR and PM, the conversion factors then remained relatively constant. With a few exceptions, the conversion factors remained below the mean staying within 1.5 %. However, for example, on 17 January 2013, four studies of the same ACR cylindrical phantom were taken at different time periods and all were above the mean. In these instances, the increase in conversion factors may be due to the use of the ACR phantom or may have been influenced by other constituents. Unfortunately, only one set of data was collected with the ACR phantom and on 17 January 2013, the option of obtaining the 20-cm cylinder phantom for a direct comparison was not available.

It is possible that room temperature fluctuations caused part of the small variations in the conversion factors. We did not measure the scanner room temperature during the study; however, it is kept within the manufacturer’s specifications, set with controls to alert the hospital’s engineering and operations for any high spikes or increased room temperature. No temperature issues were reported over the entire time period of the study.

**Fig. 4:**
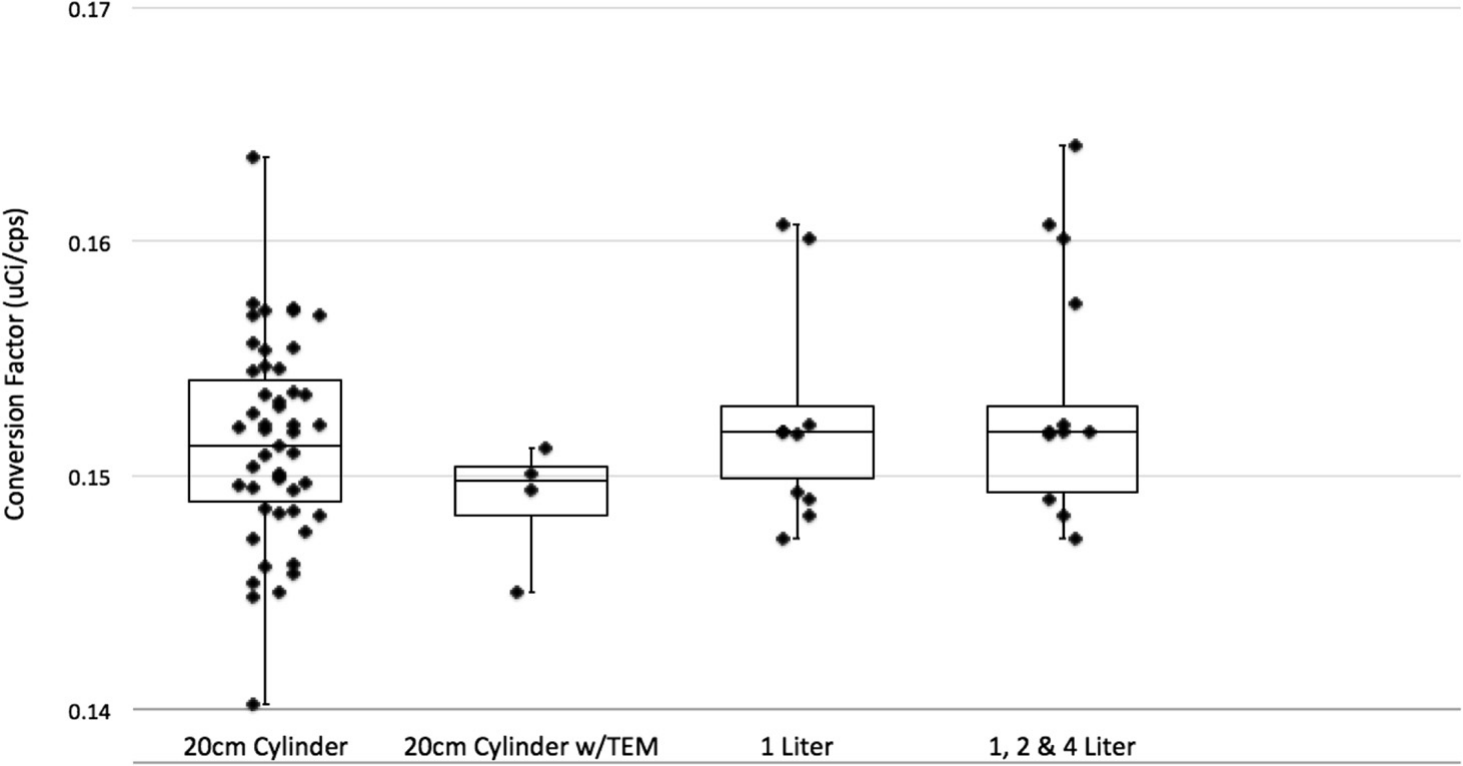
Box and whisker plot (*solid line*—median, quartiles with data points covering range), displaying the comparison of the conversion factors for the individual data sets over the course of the entire study. The data sets consist of 55 20-cm cylinder scans, four of which were with TEM, fourteen 1-L scans, two 2-L scans, and one 4-L scans

Figure 4 displays the individual components shown in graph one over the course of the study. Conversion factors are in terms of microcurie per count per second as measured in each phantom/bottle used.

The statistical analysis of the conversion factors is presented in Tables 2 and 3. The complete data set includes all the different phantoms, the 20 cm with and without TEM, the IQ and the ACR, but not the liter bottle data. The cylinder data set is the 20-cm cylinder phantom data only, without TEM. The liter bottle data set includes set 1 (the results from just the 1-L bottle) and set 2 (with the 1-, 2-, and 4-L bottles combined). Means, standard deviations, and coefficients of variation for each data set are shown in Table 2.

**Table 2:** Analysis of the conversion factor for different data sets, separating out the 20-cm cylinder phantom from the liter bottles scans. The 20-cm cylinder phantom is also analyzed with and without the tissue equivalent material. The 1-L results are also analyzed separately

Data sets	Mean (uCi/cps)	Median	Standard deviation	Coefficient of variation (%)
^99m^Tc complete cylinder data set	0.152	0.152	0.004	2.76
^99m^Tc 20-cm cylinder data (no TEM)	0.150	0.152	0.003	2.24
^99m^Tc 1-L data only	0.151	0.152	0.004	2.55
^99m^Tc combined liter data set	0.153	0.152	0.005	3.09

For an analysis of the variance (ANOVA), shown in Table 3, the following groups where compared:

Group 1: 20-cm phantom and the 1-L bottle

Group 2: 20-cm phantom and the 20 cm with TEM

Group 3: 20-cm phantom and the complete set of liter bottles (1, 2, 4)

**Table 3:** *T* test for individual two-group comparisons

Comparison groups	20-cm phantom mean	Comparison mean (1 L, 20 cm/TEM, and complete liter data set)	95 % confidence interval for difference	*p* value
20-cm phantom vs 1-L bottle	0.152	0.151	(−0.001, 0.003)	0.4125
20-cm phantom vs 20 cm phantom/TEM	0.152	0.150	(−0.002, 0.006)	0.1723
20 cm phantom vs complete liter data set	0.152	0. 153	(−0.004, 0.001)	0.1744

The comparisons did not indicate any significant differences in the mean CF values.

The complete 20 cm cylinder data set consisted of 55 scans, 4 of which are with the TEM material. The complete liter bottle data set contained 17 scans, of which 14 are 1-L bottle scans, 2 are 2-L bottle scans, and 1 is a 4-L bottle scan. The mean conversion factors for the full data set, cylinder data set, and the liter data set only vary by approximately 2 to 3 %, indicating stability.

### Precedence ^57^Co daily flood phantom

**Fig. 5:**
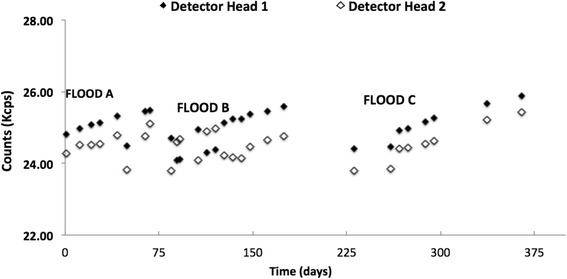
Graph of Kcounts/second collected from each detector head, 1 and 2, during a DQA for each time period—floods A, B, and C. Counts decay corrected and normalized to mean of flood A

The counts per second from the DQA ^57^Co flood scans taken on the same days as the ^99m^Tc scans are presented in Fig. 5. The graph displays the results from the three flood phantom combinations as noted in Table 1 used during the study:

- Flood A: 2011 alone
- Flood B: 2010 used with 2011
- Flood C: 2011 used with 2012

The greatest variation is seen in the time period for flood B, where detector 1 and 2 count rates vary as much as 6 %. Regarding flood A results alone and flood C alone, the detector heads vary by only approximately 3 %.

**Fig. 6:**
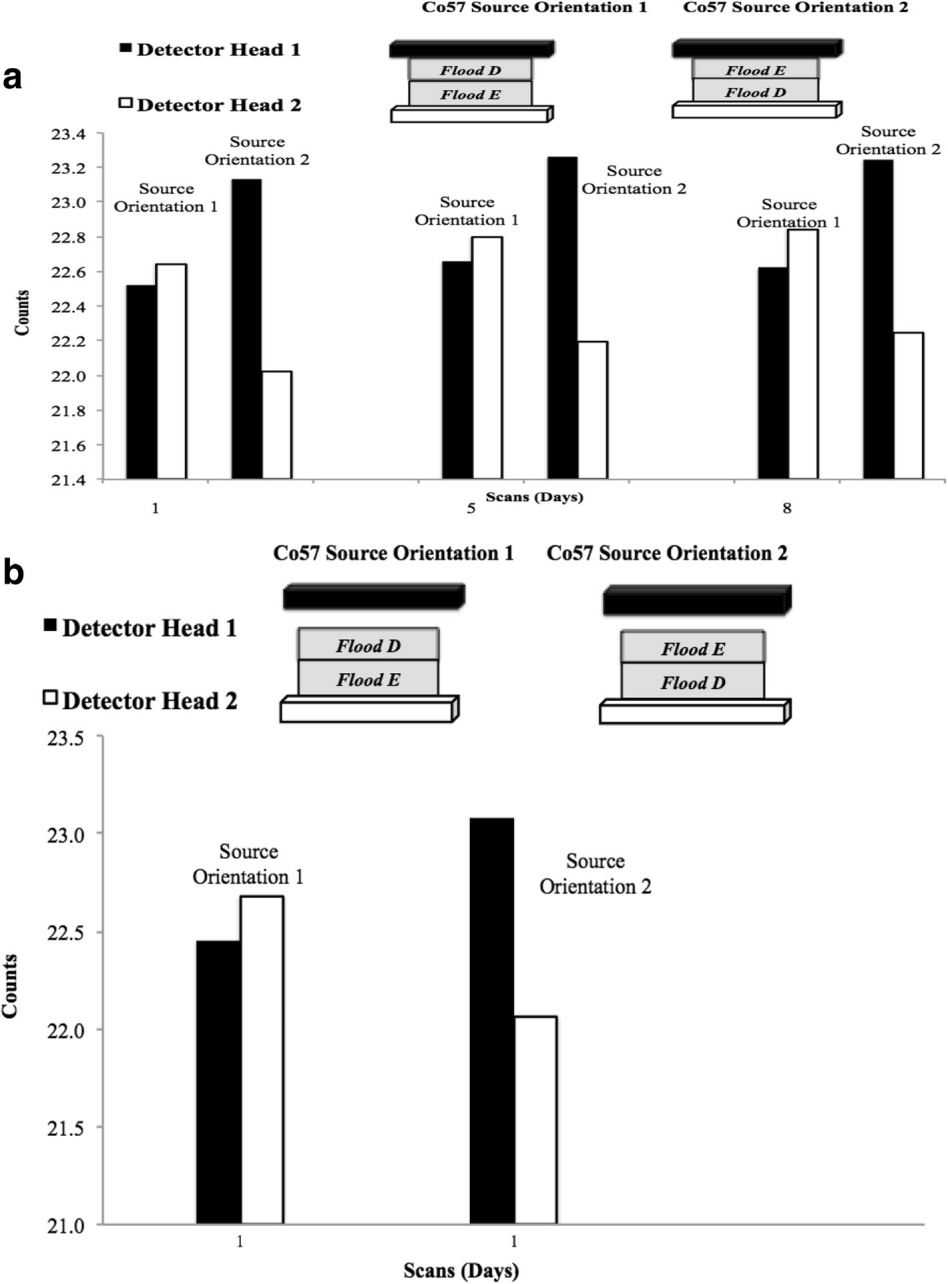
**a** Display of three sets of data (decay corrected counts/second), from detector heads 1 and 2 using two ^57^Co flood phantoms stacked in two different orientations during the DQA. The DQA was performed with each stacking orientation for that particular morning of data acquisition (both scans acquired on the same morning—days 1, 5, and 8). Note *Y-axis* does not start at zero. **b** Similar to Fig. 6a, **b** displays one set of data (counts/second), from detector heads 1 and 2 using two ^57^Co flood phantoms stacked in two different orientations during the DQA, but with detector head moved slightly away from the flood phantom. The DQA was performed with each stacking orientation for that particular morning of data acquisition (day 1 both scans were acquired). Note *Y-axis* does not start at zero

Given that flood B data showed a detector head count rate shift, the stacking of the flood source phantoms on the detectors and detector distance was examined. Three sets of data were collected during the DQA using two flood phantoms with different activity levels of ^57^Co. We were able to reproduce the variations seen in the flood B data by changing the source orientation and stacking order of the flood phantoms, shown in Fig. 2. As represented in Fig. 6a, b, we found that the stacking order correlated with the 6 % difference between detectors 1 and 2 count rate. On a separate acquisition, one data set was collected in which detector head 1 was moved away from detector head 2 creating a slightly larger distance between heads than usual. The most significant difference came when the source with less activity was placed on detector head 2; a larger gap between the detectors and flood source did not have as large an impact on the detector heads’ sensitivity.

### Precedence ^99m^Tc conversion factors compared to the ^57^Co daily flood phantom

**Fig. 7:**
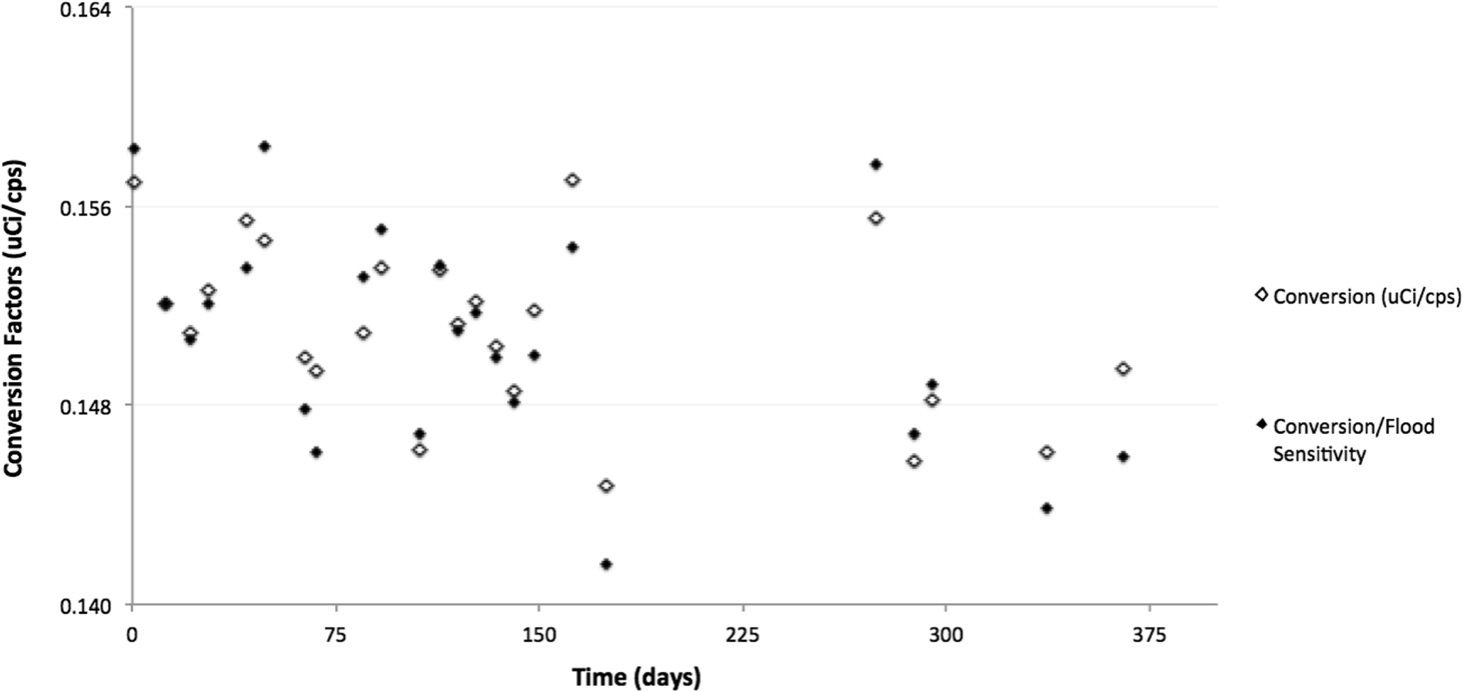
The conversion factor values compared to the conversion factors/flood sensitivity values, revealing the similar pattern

Figure 7 shows the comparison of the conversion factors to the conversion factors/flood sensitivity (uCi/cps**/**cps/normalized flood). The conversion factors and the flood data results display similar patterns. The greatest variance between the conversion factor values and the conversion factors/flood sensitivity is less than 3 %.

After separating out the data based on the time periods (flood A, flood B, flood C) for the different floods used, the conversion factors and conversion factors/flood sensitivity values vary by less than 3 %, less than 2 %, and approximately 2 %, respectively.

Table 4 below shows the mean, standard deviation, and coefficient of variation for the conversion factors/flood sensitivity.

**Table 4:** The statistical analysis of the conversion factors/flood sensitivity data set

	Mean (uCi/cps)	Standard deviation	Coefficient of variation (%)
Precedence			
^99m^Tc conversion factors/flood sensitivity	0.150	0.004	2.67

### CT images

A review of the CT images and drawn ROIs clearly indicated that the CT numbers were stable over the course of the study, eliminating the CT as a contributing factor to the CF variability.

## Discussion

### Conversion factor

We find that the scanner is relatively stable, with consistent conversion factors over the duration of the study. The maximum difference between a single conversion factor and the average conversion factor was 5 %, with the coefficient of variation of 2.76 % in the ^99m^Tc 20-cm cylinder phantom data set and a coefficient of variation of 2.67 in the ^99m^Tc conversion factors/flood sensitivity as seen in Tables 2 and 4 and Fig. 7.

In reference to Fig. 4, breaking the data down into smaller components/elements, variability in the scanner’s performance between the calculated conversion factors of the 1-L bottle compared to the 20-cm cylinder show the greatest difference to be within 10 %. Considering only the 1-L bottle and the 20-cm phantom scans that were performed on the same day, the greatest difference between the two drops to within 6 %. Conversion factors from the 2-L bottle and the 20-cm cylinder fall within 11 % of each other, while the difference between the single 4-L bottle and the 20-cm cylinder conversion factors is less than 3 %. And indicated in Table 3, the comparisons between the 20-cm phantom and the liter bottles signified that there are no significant differences in the measured mean CF values. The variability seen between the different sizes of liter bottles and the 20-cm phantom are most likely not a spatial resolution issue but can more likely be attributed to different levels of bias after scatter correction.

Furthermore, as seen in Fig. 5 and commented on below, the DQA scans were also comparatively stable with some of the variation explained by inconsistencies in the measurement process. However, the variations seen in the water-filled phantoms, displayed in Fig. 3, were greater than the variations seen in the DQA, which brings the process of filling phantoms under suspicion given that the DQA and the 20-cm phantoms were scanned under the same protocol on the same scanner. This variation is consistent with our earlier work in PET using a 20-cm water-filled phantom injected with ^18^F and a solid 20 cm ^68^Ge/^68^Ga phantom, which demonstrated that the water-filled phantom may have accounted for 1–3 % of the overall variability [14].

As a cursory check, the calculated median conversion factor value was used to recover the decay-corrected activity concentration for the 20-cm cylinder data. This check produced values in which the average percent error was within less than 3 %. Similar results were obtained with the 1-L bottle for an average percent error of less than 3 %. However, for both data sets, a few data points gave percent errors of up to 7 % between the decay-corrected activity (injected) and the recovered (measured) activity.

Figure 6a, b, the ^57^Co DQA, reveals potential areas of concern, requiring further research. First, Fig. 7 clearly shows an upward trend within all the flood data (flood A, B, and C). It is possible that the ^57^Co flood phantoms contain a longer-lived contaminant, but a more likely scenario is in the DQA protocol itself. Currently, dead-time corrections are not done in the DQA, which could help explain the upward trend seen. The other issue is the variations in sensitivity between detector heads 1 and 2. This underlying global bias from head to head will need to be corrected/accounted for in quantitative imaging.

### Continuing research

The fundamental concept of measuring/deriving the estimated amount of activity in the tissue with SPECT/CT is the same as measuring the standardized uptake value (SUV) with PET/CT. However, we feel more work needs to be done to determine the accuracy and precision of such measurements in SPECT/CT. In particular, potential errors due to scatter correction, system dead-time, collimator detector response, attenuation correction, and partial volume need further exploration. In addition, other radioisotopes, geometries, and the manufacturer’s calibration procedures need to be considered. While this paper focuses on the overall stability and calibration factors of SPECT/CT using ^99m^Tc and the DQA flood ^57^Co phantom, in order to tackle these other issues, we are currently collecting data using ^131^I, ^123^I, ^111^In, ^67^Ga, ^90^Y, and ^201^Tl, along with continued ^99m^Tc scans. These studies are being done with a variety of phantoms including an anthropomorphic phantom and varying sizes of liter cylinders with and without TEM. Furthermore, we are also looking into the validity of conversation factors for quantification along with reconstruction methods/algorithms through continued studies in a lung phantom including regions with and without injected activity.

This study demonstrates the stability of only one manufacturer’s SPECT/CT camera. Similar studies will be repeated on the other manufacturers’ SPECT/CT gamma cameras available within the University of Washington Associated Hospitals.

### Inconsistencies

Especially with complexity and variability of the phantoms used, there is a greater probability that some of the inconsistencies can be attributed to human error. Both the DQA and the activity-filled phantom require human interaction. Measuring activity from a syringe injected into a water-filled phantom creates the potential for error, from assaying pre-injection to post-injection activity levels.

Another inconsistency not usually noted is the time differences that can and do occur between the dose calibrator clock and the scanner clocks. This time difference was seen to vary from just a few minutes to as much as 10 min. For this study, clocks synced and connected to the timeserver Seiko were used.

The DQA protocol calls for a static scan using a ^57^Co flood phantom placed as close as possible to the LEHR collimators, per manufacturer’s recommendations. During this study, it was noted that the DQAs performed in our clinic involved three different phantoms and three different collimators. We also noted that the placement of the phantoms, in particular, the stacking order when using two phantoms, could result in biases. We are working on standards for a more uniform DQA, with protocols, addressing the collimators used, phantom activity and placement, energy window verification, and detector distance.

## Conclusions

This study would suggest not only that the overall performance and sensitivity of the camera are stable but also that the flood data measurements could be used to indicate when calibration factors need to be updated.

We suggest possible new protocols to augment the DQA, clearly indicating collimator usage, flood phantom placement, and distance of detectors from the phantoms and/or the development/design of a ^57^Co cylindrical phantom for placement on a stationary holder for DQA. Also, corrections during the DQA acquisition, such as dead-time corrections and the head-to-head bias, need to be addressed.

While this study demonstrated the stability of one SPECT/CT camera, more research is needed to determine the limits of quantitation for ROIs in more complex phantoms. For example, the comparisons of the results for the 1- and 2-L bottles to the larger phantoms suggest the possibility that the scatter correction for this particular image reconstruction implementation might be introducing bias in some situations.

## Abbreviations

ACR, American College of Radiology phantom; ACRIN, American College of Radiology Imaging Network; CF, conversion factor; COR, center of rotation; DQA, daily quality assurance; ESSE, effective source scatter estimation; FDA, Food and Drug Administration (USA); FS, flood sensitivity; IQ, image quality cylinder phantoms; LEHR, low-energy high-resolution; OSEM, ordered subsets expectation maximization; PET/CT, positron emission computer tomography/computer tomography; PM, preventive maintenance; SPECT/CT, single photon emission computer tomography/computer tomography; SUV, standardized uptake value; TEM, tissue equivalent material; VOI, volume of interest
